# A Case of Dry Gangrene of the Leg, Caused by Obstruction of the Common Femoral Artery—Amputation at the Middle Third of the Thigh—Recovery

**Published:** 1866-08

**Authors:** J. W. Freer

**Affiliations:** Prof. of Phys. and Surg. Anat., Rush Med. College


*A Case of Dry Gangrene of the Leg, caused by Obstruction of the Common Femoral Artery—Amputation at the Middle Third of the Thigh—Recovery.* By J. W. FREER, M. D., Prof. of Phys. and Surg. Anat., Rush Med. College.

Mr. B——, aged about 55, a robust farmer, received a kick in the groin from an unshod horse, on the 22d of April, 1866. At the time he did not consider himself seriously injured. The following quotation from a letter, written by the attending physician—Dr. Jas. B. Hawks, of Dunton, Ill.—sets forth very plainly the subsequent symptoms and results of the injury: "The foot commenced being cold the same day of the injury, and on the third day some blueish lines were observable. These gradually increased until the end of a week, when the toes had become gangrenous. Up to the third day I could detect feeble circulation in the femoral artery. At the seat of injury he did not complain of pain, and there was very little soreness. There was great difficulty in passing urine from the first to the present time—requiring the use of the catheter. The first three or four days, he could get out and in bed without assistance, and could bear his weight on the injured limb. The condition of things grew gradually worse until the time you were called."

At this time—the 9th of May—the leg was gangrenous to within about four inches of the knee. The tissues were more completely desiccated than I had ever before observed in similar cases. The general condition of the patient was very good. No symptoms of septæmia—no marked depression of the vital forces. Under these favorable circumstances, after consultation with Drs. Hawks and Hoffman, immediate amputation was considered advisable. The result was good, the stump having healed quickly and kindly.

*Remarks.*—The prominent question in this case is respecting the nature of the lesion which caused the obstruction of the femoral artery. There was no evidence of valvular disease of the heart, therefore Virchow's doctrine of disengaged fibrinous concretions floating here and there until finally arrested, as in "embolia," does not apply in this case. One can hardly conceive that a healthy artery could have been injured by a blow so slight as not to cause ecchymosis of the skin, or any apparent lesion of the soft parts; therefore, I am led to adopt the view of a condition of degeneration of the arterial walls at this point, serving to make the vessel friable, and that, in consequence, there was rupture of the internal coats, causing sufficient roughness to induce the formation of a coagulum. I have since been informed that the circulation has been re-established in the injured artery.

Mr. Rose, in the London *Lancet*, 1864, p. 60, mentions a case of embolism of the popliteal artery, following a long and fatiguing walk, which resulted in gangrene—but there was post-mortem evidence of fibrinous vegetations on the mitral valves of the heart.

There are two other cases mentioned in the *Lancet*, vol. 2, p. 228, of embolism of the femoral artery, but in each case there was heart disease with fibrous concretions on the valves.

From the length of time before complete occlusion of the vessel, (three days,) it would seem that the clot or fibrinous mass was not perfectly formed until the expiration of that time; and now that the circulation is re-established, it must have been either removed to some other part, or liquefaction must have taken place, and the dissolved particles carried into the general circulation. The latter is rather improbable.

